# Glucagon like peptide 1 receptor agonists in patients with inflammatory bowel disease: safety, clinical effectiveness and anti-inflammatory mechanisms

**DOI:** 10.3389/fgstr.2026.1864456

**Published:** 2026-08-21

**Authors:** Shannon Fernando, Joy Zhao, Kevan Josloff, Nida Zubair, Aakash Desai, Raina Shivashankar, Patricia L. Kozuch, Cuckoo Choudhary, Gary Lichtenstein, Priya Sehgal

**Affiliations:** 1 Sidney Kimmel Medical School, Thomas Jefferson, University Hospital, Philadelphia, PA, United States; 2 Department of Internal Medicine, Thomas Jefferson, University Hospital, Philadelphia, PA, United States; 3 Division of Gastroenterology, Hepatology, and Nutrition, Allegheny Health Network, Pittsburgh, PA, United States; 4 College of Medicine, Drexel University, Philadelphia, PA, United States; 5 Division of Gastroenterology and Hepatology, Thomas Jefferson University Hospital, Philadelphia, PA, United States; 6 Division of Gastroenterology and Hepatology, Perelman School of Medicine, University of Pennsylvania, Philadelphia, PA, United States

**Keywords:** glucagon-like peptide-1 receptor agonists, inflammatory bowel disease, obesity, safety, weight loss

## Abstract

**Introduction:**

The prevalence of obesity among patients with inflammatory bowel disease (IBD) continues to rise and is associated with adverse IBD outcomes and diminished treatment response. This study reviews the clinical safety and efficacy of glucagon-like peptide-1 receptor agonists (GLP-1RA) in patients with IBD and comorbid obesity and explores their theorized anti-inflammatory mechanisms.

**Methods:**

Published articles through July 2026 were identified via a PubMed search for a narrative review using terms related to GLP-1RA, IBD, safety, effectiveness, and outcomes. Data extraction focused on GLP-1RA intervention details, IBD-related outcomes and inflammatory markers, and proposed anti-inflammatory mechanisms.

**Results:**

GLP-1RA therapy promotes weight loss in patients with IBD comparable to the general population (about 16 pounds over 18 months). The most frequently reported adverse effects are gastrointestinal, often limiting treatment continuity. Most studies report no significant differences in hospitalization, surgery, steroid use or IBD medication adjustments, and some reported improvement in these outcomes. Data on endoscopic disease activity is limited. Inconsistent effects of GLP-1RA on inflammatory serum biomarkers were noted, with reductions in CRP but no consistent changes in fecal calprotectin.

**Discussion:**

Addressing obesity in patients with IBD is an emerging clinical priority. GLP-1RA therapy demonstrates efficacy in promoting weight loss, improving glycemic control, and reducing systemic inflammation, which may benefit IBD disease activity. Longitudinal studies are required to clarify the long-term impact of GLP-1RA on clinical and endoscopic outcomes in IBD.

## Introduction

Obesity has been linked to chronic inflammatory diseases due to an excess of adipose tissue that releases inflammatory mediators (1–3). As a modifiable risk factor, it has been the treatment target for various cardiovascular and endocrine diseases. The prevalence of obesity among patients with inflammatory bowel disease (IBD) is increasing, with estimates ranging from 15-40% (4–6). Obesity has also been associated with a more severe IBD phenotype, characterized by lower rates of corticosteroid-free remission, increased risk of surgery among patients receiving anti-tumor necrosis factor (TNF) alpha therapy, biologic treatment failure, and shorter intervals between flares (7–11). Obesity may represent one component of the multifactorial theory as to why 40-60% of Crohn’s disease (CD) and ulcerative colitis (UC) patients do not respond to advanced therapies (12, 13). An increasing number of IBD patients are receiving treatment for obesity or type 2 diabetes mellitus (T2DM) with glucagon-like peptide-1 receptor agonists (GLP-1RA). Despite growing usage, there is limited data and review on the safety and clinical effectiveness of GLP-1RA in IBD (14).

Regarding the relationship between IBD and obesity, a multi-center prospective study found that greater intra-abdominal visceral adipose tissue (IA-VAT) was associated with a lower likelihood of achieving corticosteroid-free or endoscopic remission (7). Specifically, CD patients with clinical or endoscopic non-response to biologics had higher IA-VAT levels compared to responders (7). This prospective study further found that biologic non-responders with elevated IA-VAT burden had increased serum interleukin-6 (IL-6) and TNF compared to responders and patients with lower IA-VAT burden, reinforcing the potential connection between visceral adiposity, systemic inflammation, and reduced biologic efficacy (7). Given this, the role of GLP-1RA therapy in optimizing IBD outcomes by targeting obesity could address a significant gap in treatment, but there are limited reviews on the summative findings on clinical outcomes and safety, which this study aims to address.

The primary aim of this review is to evaluate the safety and clinical effectiveness of GLP-1RA therapy in patients with IBD. A secondary aim is to summarize potential anti-inflammatory mechanisms of GLP-1RA therapy in IBD.

## Materials and methods

### Search strategy

A narrative review was selected given the emerging nature of the literature evaluating the use of GLP-1RA in patients with IBD. Existing studies are heterogeneous with respect to interventions and outcomes. No single comprehensive search strategy was able to capture the breadth of evidence relevant to this review, including clinical efficacy, adverse effects, disease- specific outcomes, and management considerations, which is why a systematic review was not performed.

To perform this review, published articles through July 2026 were identified through the PubMed database. Index terms (“GLP” OR “GLP-1RA”) AND (“Inflammatory bowel disease” OR “Crohn’s” OR “Ulcerative Colitis”) AND (“safety” OR “effectiveness” OR “outcomes”) were searched. This search yielded a total of 187 results. The scope of the initial review was expanded through both forward and backward citation chaining.

### Study selection criteria

Studies were excluded if their primary focus was not the relationship between IBD and GLP-1RA. Specifically, articles focused on GLP-2 analogs, short bowel syndrome, DPP-4 inhibitors, or the effects of microbiota on the GLP-1RA axis were omitted. Studies were included if they evaluated the relationship between IBD and GLP-1RA, or explored the links between metabolic syndrome and inflammation as a mechanistic rationale for the therapeutic potential of GLP-1RA in IBD.

### Data extraction

Data extraction was conducted independently by two reviewers. Variables of interest included study design, population characteristics, GLP-1RA intervention details, relevant outcomes related to IBD activity or inflammatory markers, and proposed anti-inflammatory mechanisms of GLP-1RA in IBD patients. Discrepancies were resolved through discussion.

### Quality assessment

Preprint data was excluded from the narrative review. Study relevance and methodological rigor were considered qualitatively during selection and synthesis.

## Results

### Weight loss outcomes

Several studies found that initiation of GLP-1RA therapy results in significant weight loss in patients with IBD (15). Desai et al. described comparable mean weight loss in patients with IBD and a non-IBD control cohort 18 months after initiation of semaglutide (-16 ± 13.4 pounds vs -18 ± 12.7 pounds; p = 0.24) (16). In a case series of patients with IBD on GLP-1RA, 58.3% achieved a weight reduction of at least 5% at six months, while 16.7% achieved at least 10% weight loss (17). Similarly, another cohort study reported a significant reduction in mean BMI from 34 to 31 kg/m², alongside a median total body weight loss of 8.15 kg for patients with IBD on GLP-1RA (18). Levine et al. found a significant BMI reduction (19), and Clarke et al. reported that 64.7% of IBD patients achieved at least 5% weight loss while 46.1% achieved 10% weight loss within one year (17). Sehgal et al. likewise reported 5% reduction in body weight (20). Additionally, systematic reviews of retrospective cohort studies found significant reductions in body weight (21, 22). Collectively, these studies underscore the clinical efficacy of GLP-1RA in weight loss among patients with IBD.

There is limited evidence delineating which specific GLP-1RA confers the greatest degree of weight loss in patients with IBD. Sehgal et al. found that semaglutide was associated with the greatest weight loss, with a mean reduction of -2.45 kg at 12–24 weeks (20). On the other hand, Desai et al. reported that tirzepatide was associated with a greater reduction in total body weight compared to semaglutide (16). These discordant findings regarding the relative superiority of various agents highlight the need for head-to-head prospective trials to establish definitive, evidence-based prescribing guidelines for weight management in the IBD population.

### Metabolic outcomes

Regarding metabolic outcomes, data on the effect of GLP-1RA on hemoglobin A1c and cholesterol in patients with IBD remain limited. In a sub-group of patients with IBD and T2DM, GLP-1RA was associated with a modest reduction in hemoglobin A1c of 0.3% at six months (17). However, a cohort study found no significant difference in total cholesterol or hemoglobin A1c (18). Another systematic review found that GLP-1RA decreased A1c in IBD patients, with inconsistent effects on HDL, triglycerides, LDL and total cholesterol (21). Larger studies are needed to understand the impact of GLP-1RA on metabolic outcomes in IBD.

### Safety profile

The systemic safety profile of GLP-1RA in the IBD population is largely consistent with that of the general population, although clinical utility remains constrained by gastrointestinal intolerance. A retrospective cohort study found that 11% of patients with IBD on GLP-1RA therapy experienced nausea, vomiting, and diarrhea (20). Of the 25 patients who experienced side effects, 23 patients discontinued therapy (20). Likewise, a retrospective cohort study found that 24% of IBD patients stopped GLP-1RA, and of these patients, 48% stopped therapy due to GI intolerability (23). Interestingly, one retrospective cohort study of 271 patients found no significant difference in GI adverse events exist when comparing pre- and post- treatment periods, calling into question whether experienced GI adverse events were truly secondary to GLP-1RA (24). Although ileus was mentioned as a potential GI adverse effect of GLP-1RA in the general population, a Danish cohort study including 61,927 IBD patients found that GLP-1RA was not associated with increased risk of ileus or intestinal obstruction (25). Overall, gastrointestinal adverse effects were found to be transient, dose dependent, and associated with increased weight reduction (17). In a retrospective study of 272 IBD patients on GLP-1RA, 7% of all reported adverse effects were non-gastrointestinal in nature, with no serious adverse events (23). Similarly, another study found that non-gastrointestinal adverse effects—hypoglycemia, injection site reactions, and death—each occurred in 1.1% of cases, rates comparable to those seen in non-IBD populations (26, 27). Additionally, no differences or reduced risk were observed in rates of gallbladder disease, pancreatitis, or acute kidney injury among IBD patients on GLP-1RA compared to IBD patients not on GLP-1RA (27, 28).

### Inflammatory bowel disease outcomes

#### Inflammatory biomarkers

A retrospective study evaluating the use of GLP-1RA in patients with IBD reported a significant reduction in C-reactive protein (CRP) levels from 9.0 to 1.6 mg/dL at 12–24 weeks post-initiation, independent of any change made to IBD therapy (29). However, a smaller study of 36 patients did not find any significant difference in CRP after GLP-1RA (18). For fecal calprotectin (FCP), a retrospective case series found no significant change in disease activity and no significant difference in FCP at six months after initiation of GLP-1RA therapy, also seen in a separate retrospective cohort study by Sehgal et al. (17, 29, 30).

#### Clinical outcomes

The impact of GLP-1RA on IBD-related clinical outcomes such as corticosteroid use, initiation of advanced therapy, and hospitalization remains unclear due to mixed evidence (2). A large-scale study found that GLP-1RA therapy was associated with a significant reduction in composite outcome comprised of steroid-dependence, advanced therapy initiation, IBD-related surgery, and hospitalization, which was also found in systematic reviews and retrospective cohort studies. GLP-1RA was also associated with a decreased risk of recurrent pouchitis (21, 31–35). Notably, these improved outcomes were not seen in individuals without obesity (36). Conversely, Desai et al. reported no difference in steroid use and any-cause hospitalization in IBD patients with or without GLP-1RA (16), and several other studies found no difference in rates of IBD exacerbation, hospitalization, steroid prescription, medication changes, or IBD-related surgeries after one year of GLP-1RA therapy (17, 19, 37, 38). Furthermore, Ramos et al. showed that two out of 26 patients with IBD clinically worsened with GLP-1RA therapy. One patient with CD had an increase in Harvey Bradshaw Index, and the other symptomatically worsened without increase in activity index (17).

Although the overall effect remains mixed across studies, GLP-1RA therapy has not been associated with worsened clinical outcomes overall.

Furthermore, surgical outcomes appear favorable in patients with IBD on GLP-1RA. Specifically, GLP-1RA therapy was associated with a reduction in colectomy rates in patients with UC and a lower likelihood of abdominal surgery in patients with CD (28). Notably, semaglutide was the only GLP-1RA to significantly reduce risk of IBD-related surgery in patients with CD (28).

#### Endoscopic activity

There is limited evidence surrounding the impact of GLP-1RA on IBD endoscopic disease activity. A retrospective study did not show statistically significant difference in endoscopic disease activity scores after addition of a GLP-1RA in patients with IBD (26). Interestingly, however, a case study noted endoscopic improvement after the initiation of subcutaneous liraglutide; the patient’s Mayo score decreased from 5 to 2 after 25 years of active disease (39). Large- scale prospective studies in this area are needed for better characterization.

### Suggested management strategies for patients with IBD on GLP-1RA therapy

In patients with IBD receiving GLP-1RA, treatment goals include weight loss, diabetes control, and potential optimization of disease activity and inflammation (14). There are several important clinical considerations for providers who plan to initiate GLP-1RA therapy for patients with IBD, which we outline below.

Prior to the initiation of GLP-1RA therapy, baseline IBD disease activity should be established, either through inflammatory biomarkers, CRP and FCP, or endoscopic evaluation. If a patient were to develop new- onset diarrhea or abdominal pain concerning for an IBD flare while on GLP-1RA therapy, we recommend obtaining repeat inflammatory biomarkers, CRP and FCP, and completing a thorough infectious work up to accurately delineate the etiology of acute symptomatic changes (14). If the patient develops persistent upper GI symptoms such as nausea, vomiting and dyspepsia (with concomitant normal inflammatory biomarkers), the provider should consider pursuing an upper endoscopic evaluation and gastric emptying study. If work up is normal, education for managing symptoms should be provided, such as having smaller and more frequent meals, avoiding consumption when full, avoiding spicy and high-fat foods, and moderating alcoholic and carbonated beverage intake (40). If symptoms persist, providers can also consider spacing out the GLP-1RA dosing by greater than 7 days (41).

It is also recommended that providers initiate GLP-1RA therapy at the lowest possible dose with slow titration to minimize potential adverse effects (14). Patients experiencing new onset, transient nausea can be managed with short- term antiemetic therapy. Long term, providers may consider dose reduction or prolonged dose titration (40). Any side effect requiring medical management for longer than one month may warrant dose reduction or maintenance of current dose until symptoms are tolerated (11). If patients remain unable to tolerate GLP-1RA at low doses, switching to an alternate GLP-1RA may be considered, given the variability in side effect profiles amongst individual drugs (40).

**Figure 1:**
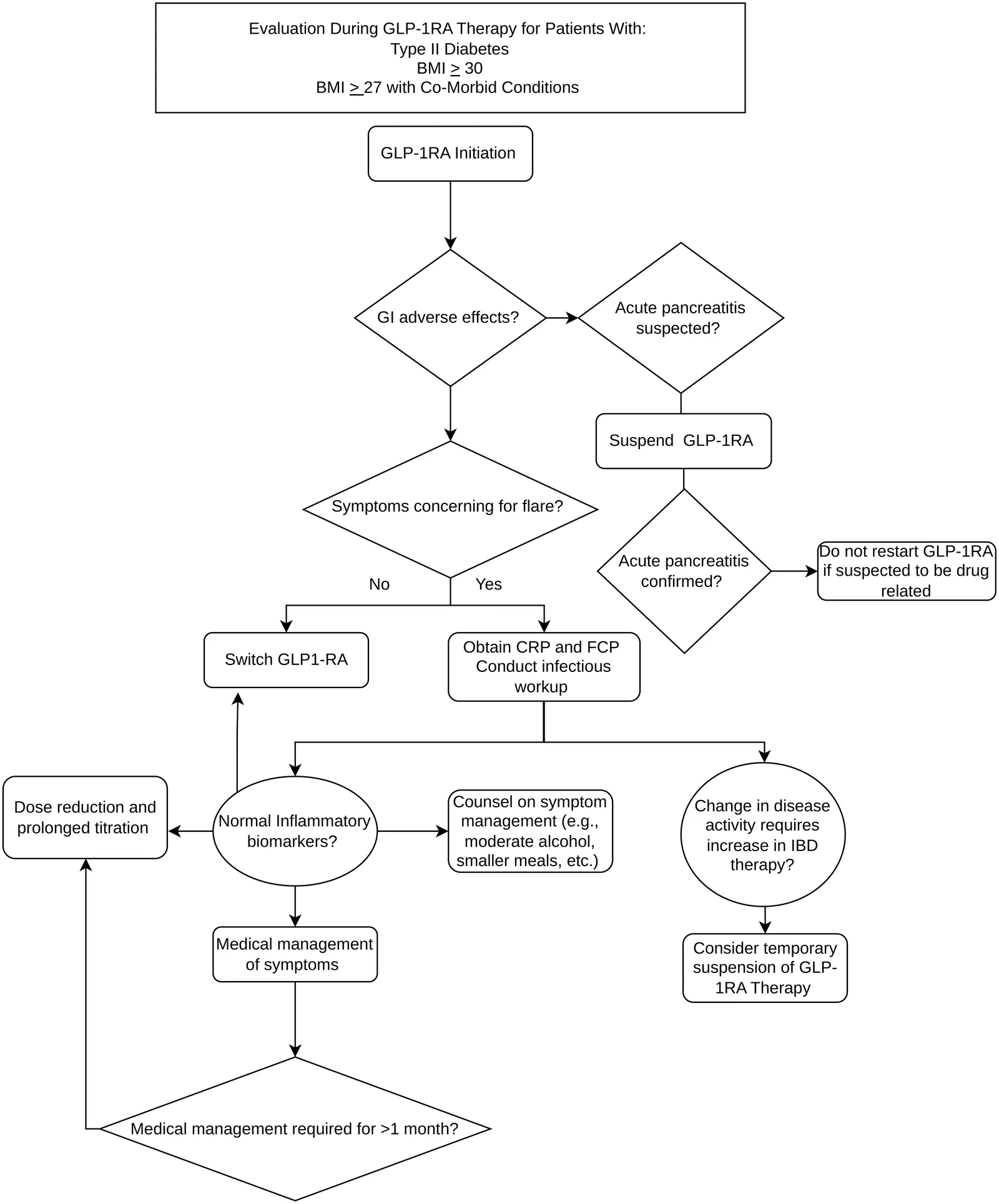
Proposed algorithm for evaluation of gastrointestinal symptoms in patients with IBD who have initiated GLP-1RA therapy (40).

There are a few instances where GLP-1RA therapy should be immediately discontinued. If the patient presents with signs and symptoms of acute pancreatitis, GLP-1RA should be immediately stopped (**Figure 1**) (40). If pancreatitis is confirmed (with no other potential etiology such as gallstones, or alcohol-induced), patients should not be restarted on GLP-1RA (40). A careful history of pancreatitis should be taken prior to initiation of GLP-1RA (14, 42). Furthermore, GLP-1RA use has been associated with the development of cholelithiasis/biliary disorders (14, 40). For patients on GLP-1RA therapy presenting with right upper quadrant abdominal pain, nausea or emesis, a thorough history should be taken, alongside objective assessments including liver function tests and abdominal ultrasound. If the patient has a new hepatobiliary issue, then GLP-1RA therapy should be stopped and appropriate management should be pursued, including cholecystectomy if necessary. If the hepatobiliary pathology is definitively managed, GLP-1RA therapy may even be able to be re-started. Furthermore, it is contraindicated to prescribe GLP-1RA to patients with a family history of medullary thyroid cancer or in patients with MEN syndrome type 2 (14). Lastly, women of reproductive age should be advised to take birth control given the unknown safety profile of GLP-1RA during pregnancy (14).

### Postulated anti-inflammatory mechanisms of GLP-1RA

There are several proposed mechanisms through which GLP-1RA therapy may reduce inflammation, and for this reason, it may serve as a potential adjuvant therapy in patients with IBD (43, 44). A proposed anti-inflammatory mechanism of GLP-1RA is inhibition of the AKT/NF-κB signaling pathway as seen in **Table 1** (45, 46). NF-kB regulates transcription of proinflammatory cytokines such as TNF-α, IL-6, IL-1β, and COX-2 (45–47). Evidence suggests that these pro-inflammatory cytokines play a role in the disruption of the intestinal barrier and inflammation seen in UC and CD (45, 46). GLP-1RA therapy may also reduce recruitment of inflammatory cells by inhibiting chemokine production from colonic smooth muscle cells (48).

**Table 1:** Proposed mechanisms underlying GLP-1RA therapeutic effects in obese individuals with IBD.

Reduction in IBD pathology implicated molecular pathways	Increased inflammation regulation	Direct anti-inflammatory effects
Inhibition of AKT/NF-κB pathway	Increased regulatory T Cells	Increased IL-10
Suppression of colonic smooth muscle cell derived cytokines	Increased IL-22 producing ILC3s	Protection against ROS
	Increased IL-33, CCL20, and MUC5B	Increased AMPK pathway signaling

Several additional mechanisms have been proposed to explain the association between GLP-1RA therapy and reduced intestinal inflammation. Exacerbated Th17 cell activation has been shown to play a role in the dysregulated immune response seen in IBD. Regulatory T cells counteract the pro-inflammatory effects of Th17, and GLP-1 analogues have been shown to induce maintenance of regulatory T cell levels (49). Furthermore, GLP-1RA therapy has also been shown to reduce intestinal inflammation, potentially through expansion of group 3 innate lymphoid cells (ILC3s) (50), a subset of cells that regulate intestinal immunity. Additional *in vivo* studies suggest that GLP-1RA upregulates IL-33, CCL20, and MUC5B expression—genes involved in mucosal healing and barrier protection (51–53). Furthermore, GLP-1RA may also promote secretion of anti-inflammatory cytokines such as IL-10 (47) and protect against reactive oxygen species (54). GLP-1 receptor agonists also increase signaling through the AMPK pathway, which leads to anti-inflammatory outcomes (47).

Ultimately, GLP-1RA therapy has the potential to serve as an adjuvant therapy in patients with IBD, acting through multiple mechanisms, including inhibiting pro-inflammatory pathways, increasing regulatory components of inflammation, and promoting anti-inflammatory pathways. These mechanisms have been primarily identified in non-human models and further research is required to ascertain the translational relevance in patients with IBD.

## Discussion

The management of inflammatory bowel disease requires a multi-faceted approach, especially as 40-60% of patients with IBD do not respond to conventional therapies (12, 13). There may be a role for optimizing patient-specific and environmental factors. Given the prevalence of obesity among patients with IBD, and its association with a more severe phenotype, obesity may represent one such modifiable factor (1). GLP-1RA therapy may therefore serve as a useful adjuvant (4). This narrative review provides a broad, updated summary on the current findings of GLP-1RA usage in IBD patients. However, there are limitations to the existing evidence, which include the retrospective nature of the studies, small sample sizes, heterogeneity in GLP-1RA agents and dosage, and lack of prospective randomized control-cohort studies to evaluate causal effect.

Nevertheless, GLP-1RA therapy has been linked to weight loss in patients with IBD (21). It is postulated that via reductions in visceral adiposity, the hormonally active component of adipose tissue, there will be reductions in the inflammatory cytokines implicated in the pathogenesis of IBD (7, 45–47). It is also theorized that reductions in mesenteric adipose tissue may decrease cytokine transcription, minimizing intestinal barrier disruption and inflammation, promoting improved disease outcome.

Various studies have evaluated the impact of GLP-1RA therapy on serum and stool biomarkers, which serve as surrogate markers for inflammation. GLP-1RA therapy has been consistently linked to reduction in CRP levels, suggesting attenuation of systemic inflammation (26, 29, 55, 56). However, this trend was not consistently seen with the stool biomarker, fecal calprotectin (29, 57). There is limited data on the impact of GLP-1RA therapy on endoscopic disease activity in IBD (19).

As reported in the literature, GLP-1RA therapy demonstrates mixed efficacy in reducing glycated hemoglobin levels in patients with IBD, although these findings are limited by a small number of studies and relatively modest sample sizes. Further prospective studies are needed to fully evaluate the impact of GLP-1RA therapy on glycated hemoglobin levels in patients with IBD.

For other IBD-related outcomes, GLP-1RA therapy has not been associated with increased risk of dose escalation of advanced therapies, initiation of steroid treatment, IBD-related hospitalizations, or IBD surgeries across multiple studies (19, 36, 37, 58, 59). Four studies observed no difference in steroid utilization for flares, whereas three studies found decreased risk of IBD-related surgeries. Two studies found a reduction in all these outcomes, including steroid-dependence, advanced therapy initiation, IBD-related surgery, and hospitalization, showing the safety and potential benefit of this therapy. Though further prospective studies are needed, there does not appear to be any association of GLP-1RA therapy with worsening of IBD disease activity.

When initiating GLP-1RA therapy in patients with IBD, it is important to establish the individual patient’s baseline disease activity to enable differentiation between drug-related adverse effects versus an IBD flare. Clinicians must exercise caution with dose escalation given the established gastrointestinal side effects, including nausea, vomiting, and diarrhea. These side effects, as well as affordability and availability of GLP-1RA therapy, have been cited as limiting factors to drug tolerance and continuity in this population. As with IBD flares, lifestyle and dietary modifications are recommended alongside pharmacotherapy for symptomatic improvement. If drug-related adverse effects persist for greater than one month, clinicians should consider dose reduction or switch of drug within GLP-1RA class. Additionally, standard precautions should be taken into consideration when initiating GLP-1RA therapy in patients with IBD as with the general population, including monitoring for acute pancreatitis, gallbladder or biliary disease, and obtaining history on MEN syndrome type 2. With this type of algorithmic approach, clinicians may feel more comfortable with prescribing and monitoring GLP-1RA therapy for patients with IBD with the goal of improving disease activity and outcomes. However, barriers including insurance coverage and patient adherence may affect its adoption in clinical practice.

In conclusion, addressing weight loss is an important component of a multi-factorial approach to the management of IBD. While the therapy has shown to be effective in weight reduction, there is also evidence of a reduction in systemic inflammation, which may contribute to long-term optimization of IBD disease activity. Future prospective randomized control-cohort studies are required to demonstrate the long term causal impact of GLP-1RA therapy on outcomes in IBD.
